# Sad music induces pleasant emotion

**DOI:** 10.3389/fpsyg.2013.00311

**Published:** 2013-06-13

**Authors:** Ai Kawakami, Kiyoshi Furukawa, Kentaro Katahira, Kazuo Okanoya

**Affiliations:** ^1^School of Fine Arts, Tokyo University of the ArtsTokyo, Japan; ^2^Emotional Information Joint Research Laboratory, RIKEN BSIWako-shi, Saitama, Japan; ^3^OKANOYA Emotional Information Project, ERATO, JSTTokyo, Japan; ^4^Center for Evolutionary Cognitive Sciences, The University of TokyoTokyo, Japan; ^5^Cognitive and Behavioral Sciences, Graduate School of Arts and Sciences, The University of TokyoTokyo, Japan

**Keywords:** sad music, vicarious emotion, perceived/felt emotion, ambivalent emotion, pleasant emotion

## Abstract

In general, sad music is thought to cause us to experience sadness, which is considered an unpleasant emotion. As a result, the question arises as to why we listen to sad music if it evokes sadness. One possible answer to this question is that we may actually feel positive emotions when we listen to sad music. This suggestion may appear to be counterintuitive; however, in this study, by dividing musical emotion into perceived emotion and felt emotion, we investigated this potential emotional response to music. We hypothesized that felt and perceived emotion may not actually coincide in this respect: sad music would be perceived as sad, but the experience of listening to sad music would evoke positive emotions. A total of 44 participants listened to musical excerpts and provided data on perceived and felt emotions by rating 62 descriptive words or phrases related to emotions on a scale that ranged from 0 (not at all) to 4 (very much). The results revealed that the sad music was perceived to be more tragic, whereas the actual experiences of the participants listening to the sad music induced them to feel more romantic, more blithe, and less tragic emotions than they actually perceived with respect to the same music. Thus, the participants experienced ambivalent emotions when they listened to the sad music. After considering the possible reasons that listeners were induced to experience emotional ambivalence by the sad music, we concluded that the formulation of a new model would be essential for examining the emotions induced by music and that this new model must entertain the possibility that what we experience when listening to music is vicarious emotion.

## Introduction

Why do we listen to sad music? Sad music induces sadness in listeners, and sadness is normally considered an unpleasant emotion that people wish to avoid. For instance, people hope to avoid misfortunes, such as the death of a loved one. However, people sometimes “lose themselves” in the beautiful sounds of sad music and even enjoy listening to it. Musicologists have been puzzled by this contradiction (Levinson, 1997), which has also been discussed in the field of philosophy. Although this contradiction has increasingly captured the attention of psychologists in recent years, few studies have investigated the issue empirically (Schubert, 1996; Garrido and Schubert, 2011; Huron, 2011; Vuoskoski et al., 2012).

In the field of emotional psychology, sadness is considered to be an affective state with negative valence. The dimensional model of emotion is one of the current models of emotion and is a circumplex model (Russell, 1980). This model suggests that emotion is a mixture of two dimensions, and various emotions can be located in a two-dimensional space with respect to coordinates of valence and arousal (Lang, 1995) or positive activation and negative activation (Watson and Tellegen, 1985). According to Russell and Feldman-Barrett (1999), emotions, including happiness, sadness, anger, fear, disgust, and surprise, can be mapped along several dimensions, such as pleasant–unpleasant and activation–deactivation. In such two-dimensional (two-axis) affective models, sadness is generally located in the third quadrant, in the same position as displeasure and deactivation emotions (Russell, 2003).

Although sadness is generally understood as negative and unpleasant in the psychology of emotion, sadness in the field of artistic appreciation may have different features or may be perceived differently. For example, in the field of drama, in which both comedy and tragedy are popular, sadness as a focus of a piece is not necessarily to be avoided. On the contrary, sadness as a central theme is fundamental to the aesthetic experience of drama. In the same manner, the type of sadness that is evoked by sad music appears to be pleasant in its own way. In fact, it is reported that some of the most beautiful and profound listening experiences are associated with sad music (Gabrielsson and Lindström, 1993). Given all of these factors, it is difficult to conclude that sadness is an unpleasant emotion when we experience it as a reaction to an artistic form such as music. Hence, the definition of sadness that is typically employed in the psychology of emotion is viewed as inappropriate for use in artistic contexts.

In this study, we attempt to promote a better understanding of sadness in relation to listening to music by investigating both perceived emotion and felt emotion. In general, the term musical emotion refers both to the perceived emotion that appears to be expressed by musical pieces and to the felt emotion that music induces in listeners. Kivy (1990) described two philosophical perspectives on musical emotion: a cognitivist perspective in which music is regarded as simply representing emotion and an emotivist perspective in which music is regarded as actually evoking emotion in listeners. In studies of musical emotion, it is essential to distinguish perceived emotion from felt emotion. Juslin (2005) viewed this distinction as crucial for three reasons: “First, the underlying mechanisms may be very different depending on the process involved. Second, measuring induced emotion is more difficult than measuring perceived emotion, and the methods must be adapted accordingly. Third, the types of emotion typically expressed and perceived in music could be rather different from the set of emotion typically induced by music” (p. 91).

Gabrielsson (2002) suggested that perceived emotion does not always coincide with felt emotion. Recently, an increasing number of studies have investigated the relationship between perceived and felt emotion in relation to music using a psychological approach (Kallinen and Ravaja, 2006; Evans and Schubert, 2008; Kawakami et al., 2013). Gabrielsson (2002) defined and categorized the relationships between perceived and felt emotion in response to music. For example, if we experience sadness when listening to sad music, then there is a “positive relationship” between our perceived and felt emotions. However, if we experience happiness when listening to sad music, then the relationship is “negative.” The latter categorization is noteworthy because it enables us to explain our regular listening behavior, in which we often enjoy sad music. According to this understanding of music and emotion, it appears that we perceive the sadness of sad music but feel both sadness and pleasure when we listen to it. Therefore, in this study, we attempt to more fully explore the relationship between perceived and felt emotions within this “negative relationship” framework.

In a recent study, Kawakami et al. (2013) investigated perceived emotion and felt emotion in an attempt to determine whether particular musical structures contribute to the difference between these two types of musical emotion. The results revealed that participants with high levels of musical experience evaluated perceived emotions for dissonant, minor-key music as unpleasant but did not experience equally unpleasant emotions in response to the same stimuli. On the contrary, these listeners experienced pleasant emotional responses to dissonance and minor-key music. Because our interest was captured by this finding that dissonant, minor-key music did not always evoke unpleasant emotional responses to musicians, despite being perceived as more unpleasant, we decided to further investigate the emotional phenomena that are associated with listening to sad music. We focused on the minor key (normally associated with sad music), which was one of the musical structures that was suspected to affect the difference between perceived and felt emotions. This focus enabled us to investigate why we sometimes “enjoy” sad music. Because the musical excerpts that Kawakami et al. (2013) used were brief and differed greatly from the music that individuals listen to on an everyday basis, it was necessary for us to resolve this issue by using existing music.

In summary, we hypothesized that felt emotion would not necessarily correspond to perceived emotion, especially in response to music in a minor key (hypothesis 1). Because listeners with substantial musical experience have been found to evaluate music in a minor key as more pleasant when rating felt emotion than when rating perceived emotion (Kawakami et al., 2013), we hypothesized that when people listened to minor-key music, those with more musical experience would feel more pleasant emotion than would be indicated by their reported perceptions of the same sad music (hypothesis 2). We sought to test this hypothesis regarding the difference between perceived and felt emotions by asking listeners to rate the perceived and felt emotions by using a collection of descriptive words or phrases. This methodology enabled us to study the subject in depth.

## Methods

### Participants

Forty-four people (25 females and 19 males) participated in our experiment (mean age of 25.3 years; *SD* = 6.6). Seventeen of these individuals were professional musicians or college students who were majoring in music (the “musician group”; *n* = 17). The other 27 participants were working people or college students who were not majoring in music (the “non-musician group”; *n* = 27).

All participants signed an informed consent agreement that indicated that they agreed to participate in our experiment. These procedures were approved by the JST (Japan Science and Technology Agency) committee.

### Materials

Three types of musical excerpts of approximately 30 s each were used. We used the following pieces as musical stimuli: (1) Glinka's La Separation (F minor), (2) Blumenfeld's Etude “Sur Mer” (G minor), and (3) Granados's Allegro de Concierto (C sharp major, but the excerpt was in G major). Because we aimed to investigate whether the perceived emotions evoked by minor-key (sad) music differed from the felt emotions evoked by the same music, we transposed Granados's Allegro de Concierto, which is normally in the key of G major, into G minor. Furthermore, because it appeared that using music in a major key would allow for a helpful comparison of the two keys for each work of music, we also transposed Glinka's La Separation into F major and Blumenfeld's Etude “Sur Mer” into G major, as the original music for those music was in F minor and G minor, respectively (see the Appendix for information regarding the scores). The excerpts were played at the following tempos: quarter note = 80 in Glinka's La Separation, half note = 72 in Blumenfeld's Etude “Sur Mer,” and quarter note = 70 in Granados's Allegro de Concierto.

We avoided selecting well-known musical pieces for use in our experiment. If well-known music had been used for the experiment, then certain participants might have had personal memories connected with this music, and the emotion evoked would thus have been influenced by those memories. Additionally, it would be difficult to support the notion that the emotions evoked by music in connection with personal memory stem solely from the music itself; rather, the emotion induced by memory-connected music may vary among individuals, for the sake of personal memories. Therefore, we asked all of the participants whether they were familiar with the musical pieces that we used in our experiment. Although the musical excerpts that we used in our experiment were derived from existing pieces, the participants indicated that they had never heard them before. Therefore, the felt emotion that the participants experienced while listening to the excerpts was regarded as resulting from the music alone rather than from personal memories.

### Self-report measures

We used 62 emotion-related descriptive words and phrases to measure both perceived and felt emotions. These descriptive words and phrases were used to measure perceived emotion by Hevner (1936) and Taniguchi (1995) and to measure felt emotion by Zentner et al. (2008). Table 1 shows the descriptive words and phrases that we used in our experiment. The participants rated the two types of musical emotions after listening to each musical excerpt by rating these descriptive words and phrases on a scale ranging from 0 (not at all) to 4 (very much).

**Table 1:** **Sixty-two emotion-related descriptive words or phrases**.

1	Happy	22	In love	43	Satisfied
2	Chills	23	Dreamy	44	Tense
3	Energetic	24	Meditative	45	Disconsolate
4	Dear	25	Stimulated	46	Admiring
5	Sentimental	26	Blue	47	Whimsical
6	Soothed	27	Agitated	48	Miserable
7	Feel like dancing	28	Moved	49	In awe
8	Sad	29	Feeling of spirituality	50	Lofty
9	Impatient	30	Triumphant	51	Determined
10	Filled with wonder	31	Sensual	52	Merry
11	Fascinated	32	Gloomy	53	Joyful
12	Fiery	33	Delicate	54	Overwhelmed
13	Tender	34	Relaxed	55	Wistful
14	Nostalgic	35	Cheerful	56	Passionate
15	Serene	36	Tearful	57	Solemn
16	Amused	37	Nervous	58	Easy passion
17	Sorrowful	38	Dazzled	59	Animated
18	Irritated	39	Inspired	60	Grave
19	Allured	40	Heroic	61	Bouncy
20	Feeling of transcendence	41	Graceful	62	Melancholic
21	Strong	42	Gay		

### Procedure

The experiment was conducted with each individual participant in a sound-insulated room. Each participant entered the room and sat across from a computer monitor. After the participant received the instructions for the experiment, s/he signed a consent form.

The participants listened to one of the three musical excerpts: 12 participants listened to Glinka's La Separation in F major and F minor, 12 participants listened to Blumenfeld's Etude “Sur Mer” in G major and G minor, and 20 participants listened to Granados's Allegro de Concierto in G major and G minor. The order of minor and major key pieces was counterbalanced across individuals. The listeners engaged in four tasks individually. In the first task, they listened to the music either in the major or minor key and rated either perceived or felt emotion using the 62 emotion-related descriptive words and phrases. In the second task, the listeners rated the same type of emotion as in the first task. If they rated perceived emotion in the first task, they also judged perceived emotion in the second task but listened to the music in a key that differed from the key in the first task. In the third task, the participants rated the remaining emotion (i.e., the emotion that they did not address in the first and second tasks) using the 62 emotion-related descriptive words and phrases after listening to the music either in a major or minor key. Finally, the listeners rated the same emotion that they answered in the third task after listening to the music in the key that differed from that of the third task. In a preliminary task occurring prior to this actual rating process, the participants had practiced rating descriptive words or phrases on a scale ranging from 0 (not at all) to 4 (very much) for both perceived and felt emotions; the participants employed the numerical keypad after listening to each of the four types of music subsequently used in the four actual rated tasks, although different music was used.

The participants listened to music emanating from a pair of loudspeakers that was located 100 cm away from them. The musical stimuli were played on a PC (DELL Vostro420) and presented via a pair of loudspeakers (FOSTEX NF-4A) through an audio mixer (EUROPACK MX 602A) at a comfortable volume level that was the same for all listeners. The participants evaluated both perceived emotion and felt emotion by responding to 62 descriptive words and phrases on a scale ranging from 0 (not at all) to 4 (very much) using the numerical keypad (ELECOM TK2-BT3H). The 62 descriptive words and phrases included both positive and negative emotions, such as happy, satisfied, joyful, sorrowful, gloomy, and miserable. These descriptive words or phrases were displayed on the monitor (iiyama Pro Lite E1902S). The data were recorded using the SuperLab 4.0 program. The time that the participants spent characterizing the two types of musical emotion depended on their individual needs; they were allowed to rate the descriptive words and phrases at their own paces.

When the participants were asked to describe their own feelings (felt emotion), they were asked to identify the emotion that they actually experienced. We used a traditional question to evaluate felt emotion: “How did you feel when listening to this musical stimulus?” In contrast, the participants were then instructed to imagine how other people would feel in response to the musical stimuli to evaluate perceived emotion. In our preliminary experiment, people with little musical experience had difficulty describing perceived emotion when they were asked to answer a traditional question: “What type of emotion does this music convey (express)?” It is plausible that, as Smith (1997) suggested, novices might “have not sufficiently developed the requisite cognitive capacities to follow music's more delicate syntactic message” (p. 253). According to Kivy (1990), emotivists believe that when people describe music in emotive terms, they are imagining how normal people feel. In addition, a person might commonly believe that her or his own feelings correspond to the emotion that is perceived in music. When the participants were asked to judge “how normal people would feel when listening to this musical stimulus,” they assumed that normal people would perceive the same emotions that they perceived themselves. In addition, the participants assumed that normal people would feel the same emotions that the participants themselves perceived in the music. For example, a participant may have the following thought process: “This music sounds sad to me and most likely to other people as well. I cannot know how other people actually feel, but my best guess is that they feel the emotion that they perceive in the music.” Thus, judgments of perceived emotion include presumptions regarding other people. Therefore, we evaluated each listener's perceived emotion by asking the following question: “How would normal people feel when listening to this musical stimulus?” This instruction was also used in the study of Kawakami et al. (2013). None of the participants reported that they could not understand the meaning of this instruction.

Asking the same participants for both types of judgments may have encouraged the participants to respond differently based on whether they were being asked about perceived emotion or felt emotion. However, we attempted to obtain answers that were not influenced by this bias by informing the participants that we did not care whether their felt emotion coincided with their perceived emotion. In previous studies in which the same participants rated both perceived and felt emotion (Kallinen and Ravaja, 2006; Evans and Schubert, 2008), the results showed that perceived emotion was similar to felt emotion in most cases. Hence, this design did not consistently bias the participants toward different judgments regarding the two types of musical emotion.

### Statistical analysis

The data that we obtained in our experiment were analyzed in two stages. First, we sorted the 62 emotion-related descriptive words and phrases by performing a factor analysis using 176 datasets: 2 (perceived/felt emotion) × 2 (major/minor key) × 44 participants. This procedure enabled us to determine the characteristics of some factors that were extracted from the analysis. Then, for each factor that was extracted through factor analysis, we conducted an ANOVA with the following design: musical emotion (perceived vs. felt) × key (major vs. minor) × musical experience (musicians vs. non-musicians) for each factor. The first two factors of the ANOVA were repeated measures, and the last factor was a between-subjects factor. The factor analysis and ANOVA were performed using SPSS for Windows (version 19.0), and *p*-values of less than 0.05 were considered significant.

## Results

### Factor analysis

The 62 emotion-related descriptive words and phrases were investigated via a factor analysis. Four factors were extracted, accounting for 62.83% of the total variance. The number of factors extracted was determined based on interpretability. An oblique rotation was performed, and the 62 emotion-related descriptive words and phrases with factor loadings are reported in Table 2. Sixteen emotion-related descriptive words or phrases, such as gloomy, meditative, and miserable, were included in Factor 1, “tragic emotion” Twenty other words or phrases, such as overwhelmed, agitated, and stimulated, were included in Factor 2, “heightened emotion” In the third factor, “romantic emotion,” there were 15 words or phrases, such as fascinated, dear, and in love. Because the 11 words or phrases in the fourth factor included merry, animated, and feel like dancing, we labeled it “blithe emotion.”

**Table 2:** **Factor loading of 62 emotion-related descriptive words or phrases**.

	**Emotion-related words or phrases**	**Factor 1**	**Factor 2**	**Factor 3**	**Factor 4**
32	Gloomy	**1.04**	−0.15	−0.05	0.20
24	Meditative	**1.04**	−0.14	−0.04	0.19
48	Miserable	**1.03**	−0.07	0.02	0.14
45	Disconsolate	**0.98**	−0.03	−0.07	0.09
26	Blue	**0.96**	−0.05	−0.10	0.17
17	Sorrowful	**0.90**	0.06	0.02	−0.04
8	Sad	**0.85**	0.06	0.05	−0.13
5	Sentimental	**0.85**	0.04	0.21	−0.07
55	Wistful	**0.85**	0.01	0.14	−0.11
62	Melancholic	**0.79**	0.06	0.15	−0.16
36	Tearful	**0.71**	0.16	0.25	−0.12
18	Irritated	**0.63**	0.05	−0.26	0.22
37	Nervous	**0.59**	0.25	−0.24	0.22
60	Grave	**0.51**	0.39	0.09	−0.24
49	In awe	**0.49**	0.36	0.24	−0.02
31	Sensual	**0.42**	0.27	0.41	−0.09
54	Overwhelmed	0.05	**0.82**	−0.11	−0.09
27	Agitated	−0.23	**0.81**	−0.08	0.05
25	Stimulated	−0.05	**0.81**	−0.27	0.03
20	Feeling of transcendence	−0.04	**0.79**	0.15	−0.11
56	Passionate	0.04	**0.76**	0.01	0.11
2	Chills	0.08	**0.74**	−0.03	−0.15
3	Energetic	−0.25	**0.73**	−0.20	0.34
21	Strong	0.12	**0.70**	−0.22	0.13
50	Lofty	−0.08	**0.69**	0.36	−0.16
40	Heroic	−0.24	**0.68**	−0.13	0.06
12	Fiery	0.18	**0.66**	−0.29	0.14
39	Inspired	0.03	**0.63**	0.07	0.13
10	Filled with wonder	0.13	**0.61**	−0.31	0.06
51	Determined	0.16	**0.60**	−0.23	−0.07
9	Impatient	0.04	**0.52**	−0.45	0.27
28	Moved	0.25	**0.50**	0.40	0.16
29	Feeling of spirituality	0.15	**0.50**	0.49	−0.07
44	Tensed	0.33	**0.48**	−0.23	−0.05
38	Dazzled	0.20	**0.47**	0.33	−0.05
57	Solemn	0.32	**0.36**	0.30	−0.32
11	Fascinated	−0.21	0.08	**0.76**	−0.01
4	Dear	0.26	−0.23	**0.72**	0.22
22	In love	0.36	−0.11	**0.71**	0.06
15	Serene	−0.04	−0.35	**0.69**	0.12
46	Admiring	−0.15	0.30	**0.69**	0.08
6	Soothed	−0.07	−0.22	**0.66**	−0.18
41	Graceful	−0.21	0.15	**0.65**	0.04
33	Delicate	0.53	−0.05	**0.64**	−0.001
13	Tender	−0.18	−0.20	**0.64**	0.15
34	Relaxed	−0.29	−0.12	**0.64**	−0.09
23	Dreamy	−0.04	0.02	**0.61**	0.29
14	Nostalgic	0.27	−0.32	**0.60**	0.18
19	Allured	−0.11	0.48	**0.50**	−0.22
43	Satisfied	−0.36	0.26	**0.48**	0.21
1	Happy	−0.37	0.05	**0.45**	0.34
52	Merry	0.03	−0.01	0.05	**0.81**
59	Animated	−0.28	0.16	−0.04	**0.70**
7	Feel like dancing	−0.11	0.08	0.12	**0.70**
61	Bouncy	−0.21	0.10	0.13	**0.69**
16	Amused	−0.21	−0.01	0.22	**0.68**
58	Easy passion	−0.05	−0.06	0.11	**0.62**
53	Joyful	−0.25	−0.01	0.25	**0.60**
35	Cheerful	−0.35	−0.01	0.25	**0.54**
42	Gay	−0.28	0.24	0.11	**0.52**
30	Triumphant	−0.34	0.34	0.05	**0.51**
47	Whimsical	0.20	0.01	−0.05	**0.47**
**Correlations among factors**		**Factor 1**	**Factor 2**	**Factor 3**	**Factor 4**
Factor 1		–	0.29	−0.30	−0.69
Factor 2			–	−0.02	0.08
Factor 3				–	0.31
Factor 4					–

Each words has four factor loadings, therefore, the highest factor loadings were bold to facilitate visualization.

### Anova

#### Factor 1 (tragic emotion)

The ANOVA revealed significant main effects for key [*F*_(1, 42)_ = 298.72, *p* < 0.001], musical emotion [*F*_(1, 42)_ = 12.14, *p* = 0.001], and musical experience [*F*_(1, 42)_ = 11.77, *p* = 0.001]. More importantly, there was a significant two-way interaction between key and musical emotion [*F*_(1, 42)_ = 26.26, *p* < 0.001].

**Figure 1:**
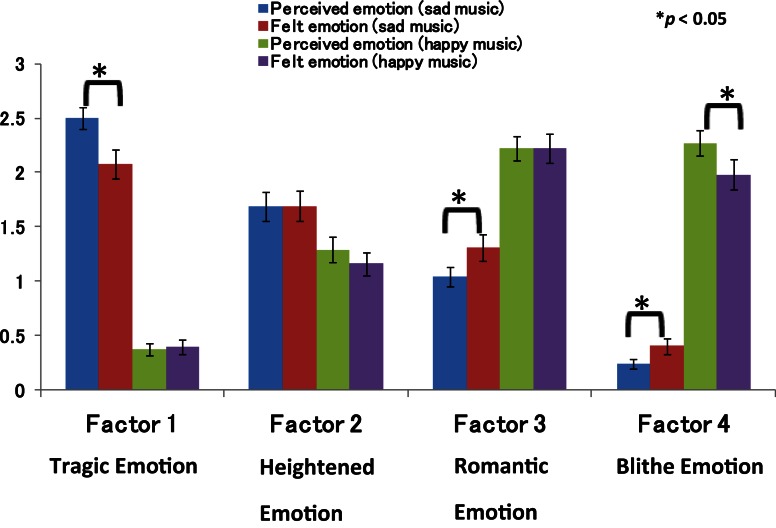
**Mean factor scores for perceived and felt emotion ratings for each factor**.

The significant two-way interaction led to a *post-hoc* analysis that indicated that the ratings of perceived emotions and felt emotions in sad music (music in a minor key) were significantly different [*F*_(1, 43)_ = 22.16, *p* < 0.001]. For tragic emotion, the perceived emotions were rated as stronger than the felt emotions (mean ratings: 2.50 and 2.08, respectively) when the participants listened to the sad music. We showed mean factor scores for perceived and felt emotion ratings for each factor as Figure 1.

#### Factor 2 (heightened emotion)

The ANOVA revealed a significant main effect for key [*F*_(1, 42)_ = 37.05, *p* < 0.001]. Heightened emotion, including feeling overwhelmed, agitated, and stimulated, was rated higher in sad music than in happy music (music in a major key), with mean ratings of 1.69 and 1.22, respectively.

#### Factor 3 (romantic emotion)

There was a significant main effect for key [*F*_(1, 42)_ = 104.18, *p* < 0.001]. More importantly, there was a significant two-way interaction between key and musical emotion [*F*_(1, 42)_ = 5.37, *p* = 0.025].

The significant two-way interaction led to a *post-hoc* analysis that indicated that the ratings of perceived emotions and felt emotions in sad music were significantly different [*F*_(1, 43)_ = 5.07, *p* = 0.03]. For romantic emotion, the felt emotions were rated as stronger than the perceived emotion (mean ratings: 1.31 and 1.04, respectively) when the participants listened to the sad music.

#### Factor 4 (blithe emotion)

There were significant main effects for key [*F*_(1, 42)_ = 193.96, *p* < 0.001] and for musical experience [*F*_(1, 42)_ = 10.02, *p* = 0.003]. More importantly, there was a significant two-way interaction between key and musical emotion [*F*_(1, 42)_ = 15.30, *p* < 0.001].

The significant two-way interaction led to a *post-hoc* analysis that indicated that the ratings of perceived emotions and felt emotions in sad music were significantly different [*F*_(1, 43)_ = 5.07, *p* = 0.03]. For blithe emotion, the felt emotions were rated as stronger than the perceived emotions for sad music (mean ratings: 0.40 and 0.24, respectively). In contrast, the analysis indicated that the ratings of the perceived emotions were higher than those of the felt emotions (mean ratings: 2.27 and 1.98, respectively) in happy music [*F*_(1, 43)_ = 8.25, *p* = 0.006].

## Discussion

### The relationship between perceived and felt emotion

We used Three-Way ANOVA to test our hypothesis that felt emotion would not necessarily correspond to perceived emotion, especially in response to music in a minor key (hypothesis 1). Our results showed a significant two-way interaction between key and musical emotion in tragic, romantic, and blithe emotion. *Post-hoc* analyses revealed that although the sad music was perceived as more tragic, the listeners did not actually experience the tragic emotion (e.g., gloomy, meditative, and miserable) to an equivalent degree. Moreover, the participants felt more romantic emotion (e.g., fascinated, dear, and in love) and blithe emotion (e.g., merry, animated, and feel like dancing) than they perceived such emotions when listening to the sad music.

In short, when the participants listened to the sad music, they indeed felt tragic emotion, but the degree to which they actually felt this emotion was lower than that for which they perceived it. Additionally, the listeners experienced romantic and blithe emotions more than they perceived these particular emotions when they listened to the sad music. The dissociation between felt and perceived emotion is selective for three out of four of the found factors. In view of our results, we consider hypothesis 1 to be confirmed.

With respect to the happy music, perceived emotions were rated higher than felt emotions for blithe emotions including feeling merry, feeling animated, and feeling like dancing. There were no significant differences between the perceived and felt emotions for tragic emotion, heightened emotion, and romantic emotion.

For the sad music, the perceived emotions were rated higher than the felt emotions for tragic emotion. For the happy music, the same was true for blithe emotion. In general, it appears that perceived emotions may be rated higher than felt emotions for emotional categories that have a strong association with a certain key.

### The effects of musical experience

The difference between felt and perceived emotions was not affected by musical experience in this study. Our hypothesis was that when people listened to minor-key music, those with more musical experience would feel more pleasant emotions than they would perceive with respect to the sad music (hypothesis 2), but this hypothesis was not supported by our results.

In contrast, Kawakami et al. (2013) found a significant difference between perceived and felt emotions only for participants with high levels of musical experience when they listened to short minor-key musical stimuli. Musically trained people rated perceived emotions as more unpleasant than felt emotions when they were exposed to these musical stimuli. Additionally, these individuals experienced fewer unpleasant feelings and more pleasant feelings when they listened to music in a minor key that featured dissonance. These results suggested the possibility that people with high levels of musical experience may be influenced by an “aesthetic” assessment of the sad music when rating their felt emotion. Moreover, the musically trained people appeared to be more tolerant of dissonance than were laypersons; as noted by Webster and Weir (2005), individuals with more musical experience are likely to have been exposed to minor-key music more often than people with less musical experience. This exposure may lead to familiarity, which could in turn generate more positive ratings.

However, in the current study, we did not find that musical experience had an effect on perceived vs. felt emotions when the participants listened to the sad music. Independent of their musical experience, the listeners felt less gloomy, meditative, and miserable as well as more fascinated, dear, in love, merry, animated, and inclined to dance when they listened to sad music compared with their actual perceptions of the same music. The reason that musical experience was not important in the difference between the perceived and felt emotions for the sad music could lie in the musical stimuli that we used. Because the musical stimuli used by Kawakami et al. (2013) consisted of only a few measures (from one to four measures), they lacked ecological validity. By contrast, the musical stimuli in this experiment were excerpts from existing musical pieces (from nine to 19 measures). Therefore, the listeners were able to capture more information about the musical structures for the music to which they were listening than they was allowed by the musical stimuli in the experiment by Kawakami et al. (2013). As a result, the participants may have been able to react to the aesthetic aspects of the sad music, regardless of their musical experience, leading to the disappearance of the difference between the musicians and non-musicians regarding perceived and felt emotion in relation to the sad music. Given the familiar phenomenon that people, regardless of their musical experience, can enjoy sad music in everyday life, it seems natural that we did not find a difference between the musicians and non-musicians regarding perceived and felt emotions.

### Listening to sad music induces ambivalent emotions

In the psychology of emotion, as a rule, sadness is classified as unpleasant in an evaluative dimension (unpleasant–pleasant). If an emotion with properties that are similar to those of a sad perceived emotion were evoked in listeners, then they would experience more unpleasant emotion when they listened to the sad music. If sad music actually evokes only unpleasant emotions in listeners, then why do people listen to sad music? Green et al. (2008) found that although minor-key music was judged to be sadder than major-key music, the former was rated as more likeable than the latter. It is peculiar that people appear to love stimuli that induce only unpleasant emotions.

Although musicologists have experienced difficulties in attempting to explain why people listen to sad music (Levinson, 1997), the findings from this study may be able to provide possible answers to the question of why people listen to sad music. In accordance with an earlier study that revealed minor-key music to be perceived as sad (Hevner, 1935), the perceived emotions in this study were rated as more unpleasant (e.g., more gloomy, meditative, and miserable) than the felt emotions with respect to the sad music. Additionally, the participants actually experienced gloomy, meditative, and miserable emotions when they listened to the sad music, although the degree of felt emotions was lower than that of perceived emotions. In addition, participants experienced not only sad emotion but also heightened romantic and blithe emotion when they listened to the sad music. It is assumed that listeners were in an ambivalent emotional state to listen to the sad music. Because sad music elicits both sad and pleasant emotions in listeners, people may choose to spend a significant amount of time listening to sad music and even enjoying it (Schubert, 1996; Vuoskoski et al., 2012).

Against this backdrop, why do we experience ambivalent emotions when we listen to the sad music? The positive relationship that was suggested by Gabrielsson (2002) appears to be the simplest explanation for the relationship between perceived and felt emotions to explain how sad music could evoke negative emotions in listeners—notably, most previous studies had not distinguished perceived emotion from felt emotion. More precisely, the results showing that people experience ambivalent emotions in response to sad music demonstrate that perceived and felt emotions do not coincide when people listen to sad music. Thus, we must question why this relationship has emerged. In this respect, we consider the following possibilities from three approaches.

### Sweet anticipation

Tragic emotion including sadness may actually be unpleasant, in accordance with the field of emotion psychology. In fact, we might experience unpleasant emotions when we listen to sad music; however, we simultaneously experience pleasant emotions. Therefore, we feel comfortable when we listen to sad music. When we listen to music, we are apt to anticipate what is coming next (Meyer, 1956). According to this musical expectancy theory, listeners experience emotion because of violation, delay, or confirmation of their expectations regarding the continuation of the music. In a previous study, Huron (2006) examined the reasons that people experience positive emotion when listening to music. He believed that the prediction effect would affect the emotion that listeners experienced. That is, listeners experience positive feelings when a future event is successfully predicted. If the sound that listeners have expected is heard, thereby confirming their expectations, then listeners experience positive emotions (“sweet anticipation”) as a result of this process. Even if listeners experience negative emotions when listening to sad music, sweet anticipation might still allow them to feel positive emotions.

### Re-evaluation when listening to sad music in the context of art

When we consider the process of evoked emotion in the context of the two-factor theory of emotion (Schachter and Singer, 1962) and the cognitive-mediational theory developed by Lazarus (1966, 1993), the role of the appraisal process in the elicitation of emotion becomes important. According to Lazarus (1966), people must be able to understand their own situations to experience emotion. In the case of musical emotion, the situation of music listening could be regarded as a target for cognitive appraisal. That is, the context in which people listen to music is likely to be important in cognitive appraisal. Whether the emotion that we perceive is pleasant or unpleasant, we might experience the emotion as pleasant through a cognitive appraisal process that recognizes that an emotion is evoked in an aesthetic context. Consequently, even if the music itself is perceived as negative, and negative emotion is aroused in listeners in part, we have a tendency to experience ambivalent emotions by concurrently feeling pleased by virtue of our cognitive appraisal. Perhaps we initially experience negative emotion, such as sadness, and subsequently experience pleasant emotion because of the rewarding effect of enjoying art (Koelsch, 2012). Thus, the experience of listening to sad music may ultimately elicit pleasant emotion.

### Vicarious experiences and emotions in relation to art

The two approaches above assume that an unpleasant emotional experience is initially induced in listeners when listening to music. First, is the “unpleasant” emotion (e.g., sadness) experienced through art actually unpleasant at all? As Eerola and Vuoskoski (2011) noted, although sadness is generally considered to be an unpleasant emotion, sadness in the context of music might not be classified as unpleasant in an equivalent manner. In the context of art, emotion-evoking processes may differ from those of day-to-day emotions. Thus, the sadness that we experience while listening to sad music may differ from that which we experience in our daily lives, as Scherer (2004) proposed when discussing the distinction between goal-oriented utilitarian emotion and aesthetic emotion. In fact, some researchers have denied that music can induce common “everyday emotions” (e.g., sadness, happiness, and anger; Kivy, 1990; Konečni, 2003; Scherer, 2003). Noy (1993) also noted that “the emotion evoked by music are not identical with the emotion aroused by everyday interpersonal activity” (p. 126).

Given this reasoning, what is the difference between day-to-day emotions and emotions that are evoked by music? In the emotions that we experience in daily life, an actor who experiences emotions has a direct relationship with the object or situation by which the emotion is aroused. In contrast, when we listen to music, a person is safe from any threat or danger that the music represents (Zentner et al., 2008). Thus, the emotions that we experience when we listen to music may be characterized as vicarious property. When we feel a certain type of emotion when listening to music, there is no objective or situation that acts as a cause to induce emotion as in everyday life. Rather, the composer, performer, or music itself that expresses emotion may be the entity that enjoys the direct relationship with the originating situation. The felt emotion of music can be regarded as a vicarious emotion if we conceptualize our experience of the emotion, which originated with the composer, performer, or music itself, as occurring through a mechanism such as sympathy. In contrast to the emotions of everyday life, such a vicarious emotion would not be accompanied by any essential pleasantness or unpleasantness that provides incentives to approach or avoid it.

Here, it should be noted that extra-musically evoked emotions might not fall under the definition of vicarious emotion. When music is connected with a personal memory, such as a lost love or someone's death, the listener may experience a sad emotion that is accompanied with suffering, similar to how the emotion is experienced in everyday life. However, the emotion that such a listener experiences stems from memory rather than solely from the music. In this case, music triggers the creation of the emotion, and memory is considered to be the agent directly inducing the emotion. Even in the case in which music is connected with autobiographical memory, listeners often appear to experience positive emotion (Janata et al., 2007).

The sad emotion that is induced when we listen to sad music that is not accompanied by extra-musical factors would be regarded as vicarious and can be pleasant. Because the danger that is associated with listening to sad music does not pose a direct threat to us, listeners are able to trust and enjoy the listening process. In addition, by adding sweet anticipation or a cognitive appraisal of the aesthetic experience as described above, listeners can experience the emotional quality of sadness while simultaneously experiencing ambivalent emotions accompanied with pleasant or unpleasant emotions. This experience allows listeners to feel pleasure regardless of whether a piece of music expresses sadness or happiness (Salimpoor et al., 2009). In this manner, the arts appear to provide simulated experiences of emotion in which one can regard the world in a setting of guaranteed emotional safety.

### A new model of musical emotion

Because music is assumed to have no real implications for an individual's well-being (Zentner et al., 2008), sad emotions induced by listening to music may be vicarious ones. Moreover, unlike emotions in everyday life, emotions that are evoked by music cannot elicit unpleasant experiences. Thus, it seems unsuitable to consider sad emotions through music in a traditional emotion model, such as the two-dimensional affective model with two axes (e.g., pleasant–unpleasant and aroused–sleepy) because such a model assumes everyday-life emotions—i.e., emotions elicited in non-musical contexts. Such traditional models cannot represent the vicarious nature of musical emotions. Therefore, the adoption of a new model appears essential for understanding musical emotion.

**Figure 2:**
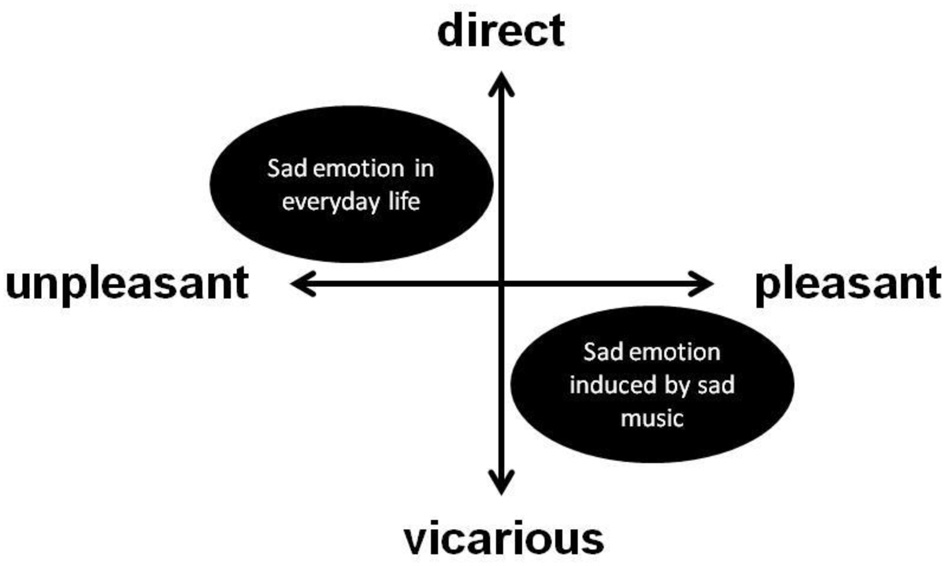
**Pleasant–unpleasant, direct–vicarious model**.

Figure 2 shows the two-dimensional affective space (pleasant–unpleasant, direct–vicarious). The horizontal axis shows the emotional evaluation of experienced emotion, and the vertical axis shows the relationship with the object causing the emotion. The relationship axis represents the manner in which an individual relates to the stimuli that elicit the emotion, which is exactly what differentiates the emotions of everyday life from those that occur when listening to music; this axis frames “direct” and “vicarious” as polar opposites. Thus, emotions that are experienced in everyday life would be located in the first and second quadrants in Figure 2. The emotions that are felt when listening to music are considered below.

As defined in an existing theory of emotion, emotions experienced in everyday life are distributed widely across the first and second quadrants—i.e., from pleasant to unpleasant. For example, sad emotions in daily life would be mapped in the second quadrant, as shown in Figure 2. In contrast, felt emotions in music, including both sad and happy music, would be located primarily in the fourth quadrant. Given that the emotions elicited by music are vicarious, they cannot include unpleasant experiences and would even be almost pleasant because of the cognitive processes associated with listening to music. Therefore, the sad emotions that listeners feel when listening to sad music could be mapped in the fourth quadrant, as shown in Figure 2.

Although this model would be insufficient for completely describing both the emotions of everyday life and of music, it systematically reflects the difficulty that exists when attempting to apply the framework used to describe everyday emotion to emotions that are relevant to aesthetic experiences. In the future, it will be necessary to construct an elaborated, comprehensive model that would account for both the emotions of everyday life and those of the musical context.

## Future issues and perspectives

Finally, we would like to consider some future issues and perspectives. In this study, we asked participants to rate perceived and felt emotions by using 62 emotion-related descriptive words after listening to the music. Although it did not take listeners long to rate the emotions, there was a possible issue about memory. Specifically, the emotions of listeners might have been diminished while they were answering emotional words and phrases. Therefore, a better way to measure emotions would be to use a continuous measurement. Indeed, developing a measure to assess perceived emotions continuously has been of particular interest in recent studies (Schubert, 1999; Nagel et al., 2007).

In addition, we have to consider the instructions given to the participants. To measure perceived emotions, we used the following prompt: “How would normal people feel when listening to this musical stimulus?” This instruction might depend on the ability to make assumptions of other people's mental states. This ability would involve empathy, which has been studied in the field of neuroscience and developmental psychology. Therefore, the psychological process underlying the perception of emotion by using our instruction should be verified in detail in the future. However, we believe that the instruction to measure perceived emotion in this study is reasonable, as explained in the fourth paragraph of the Procedure section.

Although perceived and felt emotions were found to differ behaviorally when people listened to sad music, further investigation is needed to confirm these behavioral data. It is possible that that because individuals had to rate each heard piece by using 62 emotion-related descriptive words, the evaluation was based more strongly on memory and cognition than on “hot” emotional appraisal. In another study, Huron investigated the relationship between feelings of pleasure and prolactin concentrations in response to sad music. The author found that high prolactin concentrations were correlated with pleasurable sadness evoked by sad music while low prolactin concentrations were correlated with unpleasant sadness (Huron, 2011). In the future, measuring other indicators, such as physiological responses and neural network would be essential to determine the reason behind the pleasant emotions felt in response to sad music.

## Conclusion

In this study, we examined the question of “why people listen to sad music” by dividing musical emotion into perceived emotion and felt emotion. We hypothesized that felt and perceived emotion may not actually coincide in this respect: sad music would be perceived as sad, but the experience of listening to sad music would evoke positive emotions. The results revealed that although sad music was perceived to be more tragic, listening to sad music actually induced participants to feel more romantic, blither, and less tragic. Thus, the participants seemed to experience ambivalent emotions when listening to sad music. This is possibly because the emotion induced by music is indirect, that is, not induced by personal events, which somehow induces participants to feel pleasure as well.

### Conflict of interest statement

The authors declare that the research was conducted in the absence of any commercial or financial relationships that could be construed as a potential conflict of interest.

## Figures and Tables

**Figure:**
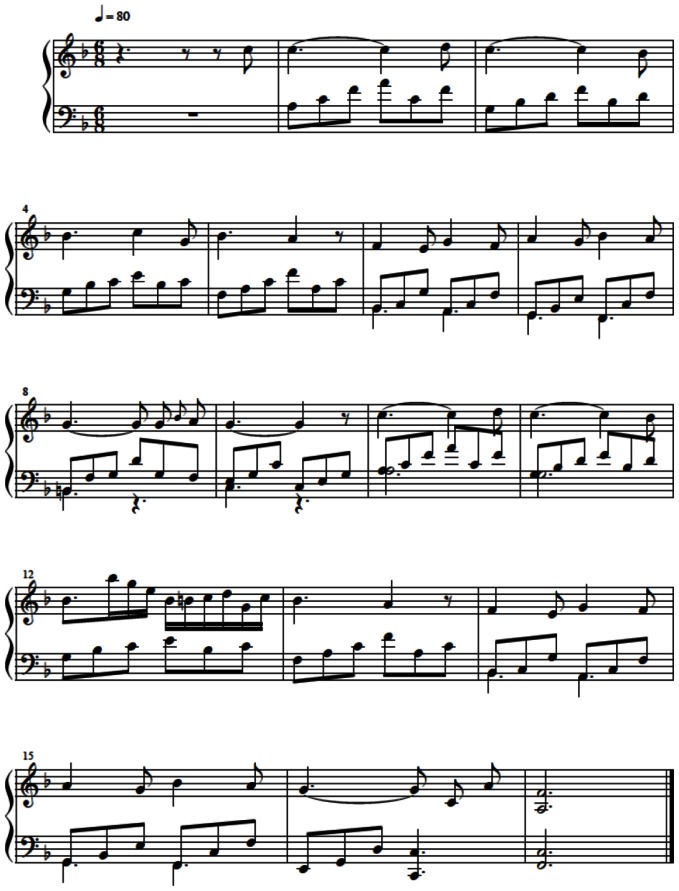
**Glinka: *La Separation* (F major)**.

**Figure:**
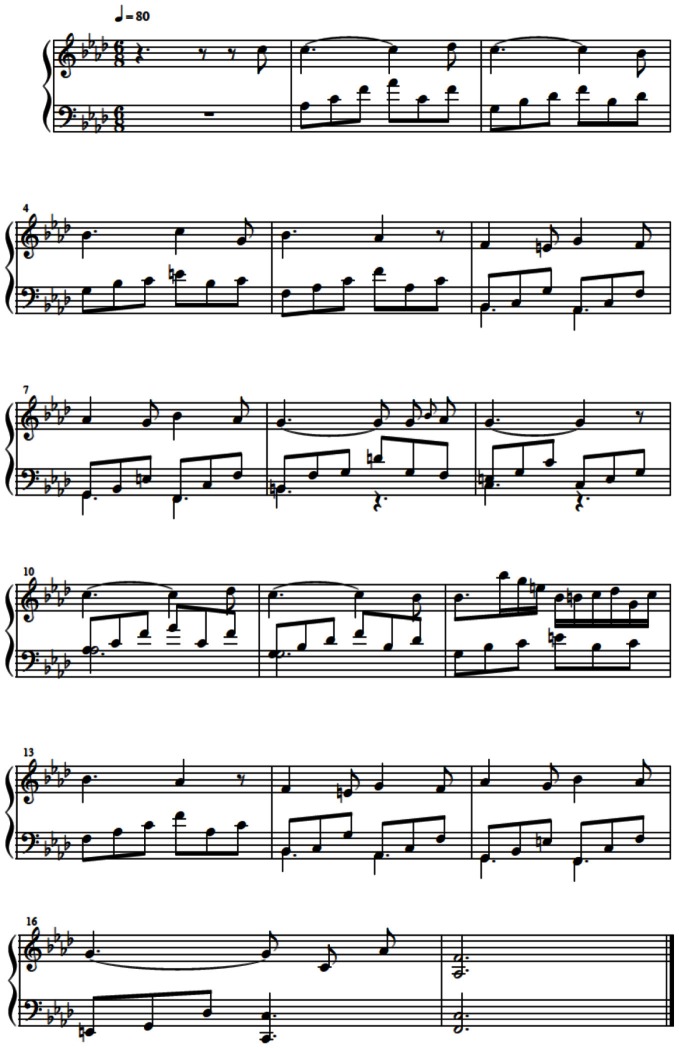
**Glinka: *La Separation* (F minor)**.

**Figure:**
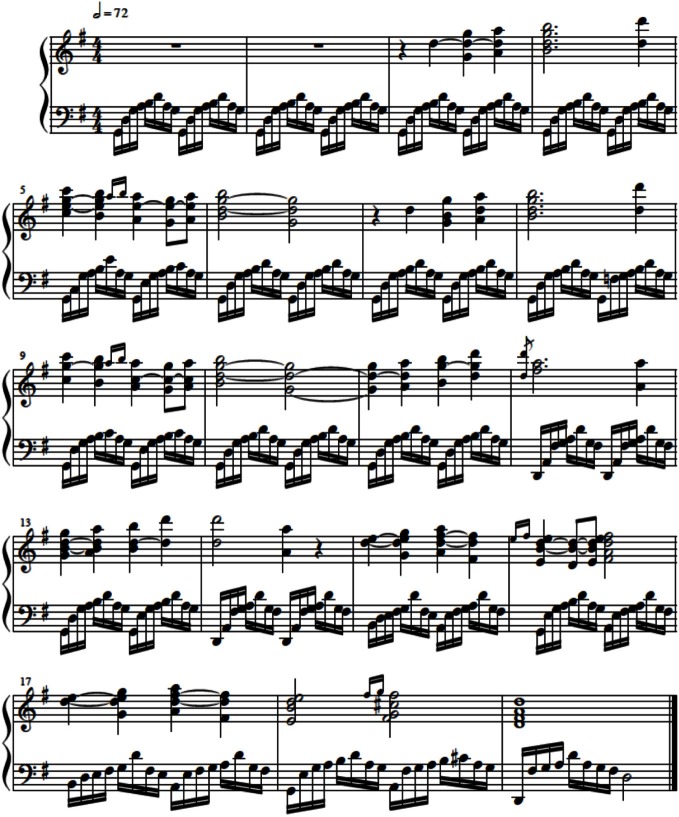
**Blumenfeld: *Etude “Sur Mer”* (G major)**.

**Figure:**
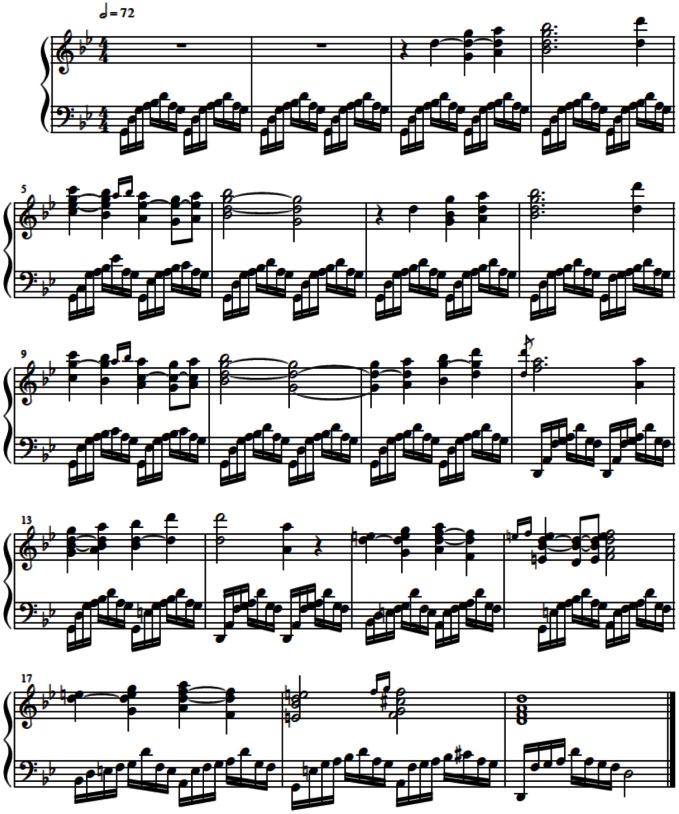
**Blumenfeld: *Etude “Sur Mer”* (G minor)**.

**Figure:**
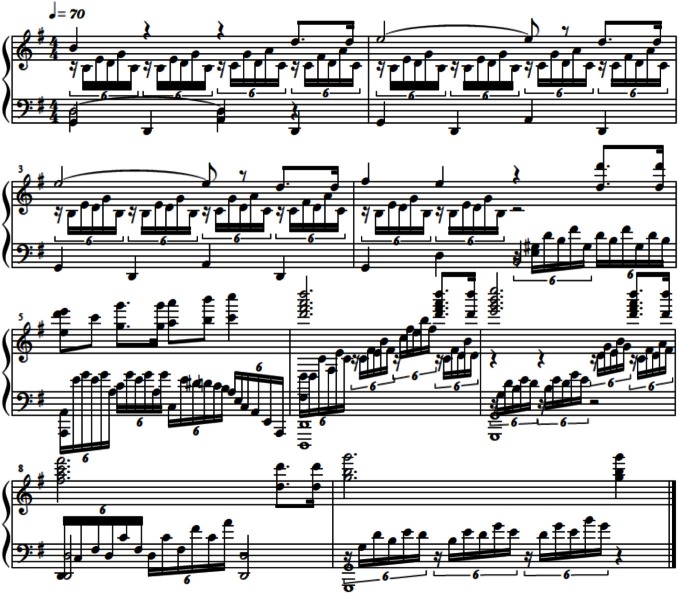
**Granados: *Allegro de Concierto* (G major)**.

**Figure:**
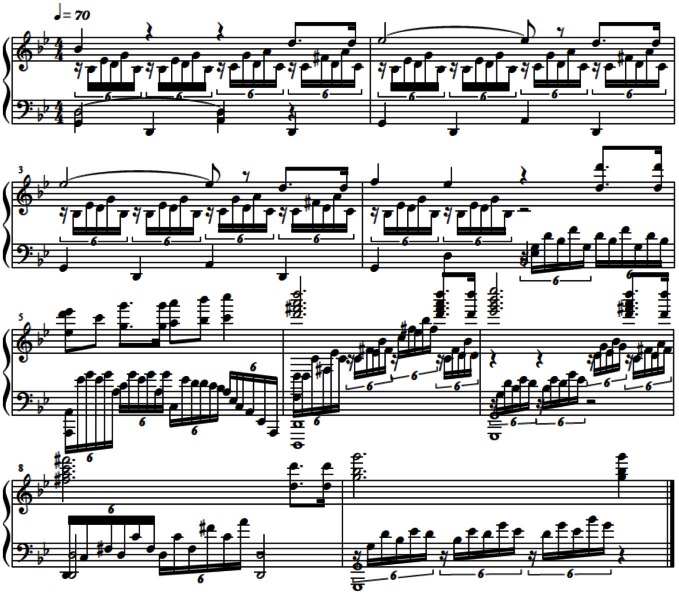
**Granados: *Allegro de Concierto* (G minor)**.
